# Optimal Route for Mesenchymal Stem Cells Transplantation after Severe Intraventricular Hemorrhage in Newborn Rats

**DOI:** 10.1371/journal.pone.0132919

**Published:** 2015-07-24

**Authors:** So Yoon Ahn, Yun Sil Chang, Dong Kyung Sung, Se In Sung, Hye Soo Yoo, Geun Ho Im, Soo Jin Choi, Won Soon Park

**Affiliations:** 1 Department of Pediatrics, Samsung Medical Center, Sungkyunkwan University School of Medicine, Seoul, Korea; 2 Samsung Biomedical Research Institute, Samsung Medical Center, Sungkyunkwan University School of Medicine, Seoul, Korea; 3 Biomedical Research Institute, MEDIPOST Co., Ltd., Seoul, Korea; Rutgers - New Jersey Medical School, UNITED STATES

## Abstract

Recently, we showed that intracerebroventricular (IC) transplantation of human umbilical cord blood (UCB)-derived mesenchymal stem cells (MSCs) significantly attenuates posthemorrhagic hydrocephalus (PHH) and brain damage after severe IVH in newborn rats. This study was performed to determine the optimal route for transplanting MSCs for severe IVH by comparing IC transplantation, intravenous (IV) transplantation, and IV transplantation plus mannitol infusion. Severe IVH was induced by injecting 100 uL of blood into each ventricle of Sprague-Dawley rats on postnatal day 4 (P4). After confirming severe IVH with brain magnetic resonance imaging (MRI) at P5, human UCB-derived MSCs were transplanted at P6 by an IC route (1×10^5^), an IV route (5×10^5^), or an IV route with mannitol infused. Follow-up brain MRIs and rotarod tests were performed. At P32, brain tissue samples were obtained for biochemical and histological analyses. Although more MSCs localized to the brain after IC than after IV delivery, both methods were equally effective in preventing PHH; attenuating impaired rotarod test; increasing the number of TUNEL-positive cells, inflammatory cytokines, and astrogliosis; and reducing corpus callosal thickness and myelin basic protein expression after severe IVH regardless of mannitol co-infusion. Despite the superior delivery efficacy with IC than with the IV route, both IC and IV transplantation of MSCs had equal therapeutic efficacy in protecting against severe IVH. These findings suggest that the less invasive IV route might be a good alternative for clinically unstable, very preterm infants that cannot tolerate a more invasive IC delivery of MSCs.

## Introduction

Intraventricular hemorrhage (IVH) is a common and serious disorder in premature infants[1]. As the risk and severity of IVH correlates with the extent of immaturity[2], advances in perinatal medicine that have improved the survival of very preterm infants have increased the number of cases of preterm infants at high risk for developing IVH[3]. Severe IVH and the ensuing posthemorrhagic hydrocephalus (PHH) cause brain injury, and finally results in increased mortality and long-term neurologic morbidity, such as seizure, cerebral palsy, and developmental retardation in survivors[4–6]. Although the pathogenesis of brain injury and PHH after severe IVH has not been clearly elucidated, these conditions might result from inflammatory responses caused by blood contact and blood products in the subarachnoid space[7, 8]. Since no effective treatment is currently available to prevent PHH or attenuate brain damage after severe IVH in preterm infants, a new therapeutic modality to improve the prognosis of this intractable disease is urgently needed.

Recently, we showed that intracerebroventricular (IC) xenotransplantation of human umbilical cord blood (UCB)-derived mesenchymal stem cells (MSCs) significantly attenuated brain damage and PHH after severe IVH in immune competent newborn rats. This neuroprotective mechanism was primarily mediated by the anti-inflammatory effects of the transplanted MSCs[9]. Overall, these findings suggest that transplanting human UCB-derived MSCs could be a novel therapy for severe IVH in preterm infants.

Determining the optimal route to transplant MSCs is essential to translate these experimental results to clinical trials. Using the minimally invasive and practical technique of systemic intravenous (IV) transplantation of MSCs has distinct therapeutic advantages in patients with severe IVH. Furthermore, as IVH usually develops within the critical time period of 3 days after birth[10], some very preterm infants in a clinically severe condition might not tolerate an invasive local IC injection of MSCs. Some animal data suggest that the IV route is not optimal for treating local brain lesions because transplanted cells might be retained in other organs such as the pulmonary capillaries, liver, spleen, or kidneys[6, 11, 12]. Transplanted cells might also have limitations crossing the blood brain barrier (BBB), resulting in inferior stem cell delivery efficiency compared with local IC administration[13, 14]. Combined treatment with a BBB permeabilizer (mannitol) might facilitate cells passing through the BBB and enhance the efficacy of systemic IV transplantation of MSCs[15]. In the present study, therefore, we tried to determine the optimal route of MSC transplantation after severe IVH in newborn rats by comparing the efficacy of three methods of MSC administration: local IC, systemic IV, and systemic IV with a BBB permeabilizer (mannitol).

## Methods

This study was approved by the Institutional Review Board of Medipost Co., Ltd., Seoul, Korea (Permit Number; MP-IRB-20060821-1, 2008-10-1). All experimental protocols were approved by the Institutional Animal Care and Use Committee of Samsung Biomedical Research Institute (Permit Number: 2013121800), and the study followed institutional and National Institutes of Health guidelines for laboratory animal care.

### Cell Preparation

As previously reported, UCB was collected from umbilical veins after neonatal delivery with written informed consent from the mother, and MSCs were isolated and cultivated [16–18]. Details of expression markers and antigens are described in our previous reports [9, 18–22]. Human UCB-derived MSCs differentiated into various cell types such as respiratory epithelium, osteoblasts, chondrocytes, and adipocytes with specific in vitro induction stimuli[16, 17, 20, 23]. We confirmed the differentiation potential and karyotypic stability of the hUCB-MSCs up to the 11th passage. MSCs were labeled with micron-sized paramagnetic iron-oxide (MPIO) to confirm their location and track donor cells by follow-up T2*W brain MRI[9, 19].

### Animal Model

All animal procedures were performed in an AAALAC-accredited specific pathogen-free facility. Newborn Sprague-Dawley rats (Orient Co., Seoul, Korea), those reared with their dams in the standard cage, 50 liter Plexiglas chamber, were used in this study. Dam rats could assess to water and laboratory chow freely, and were maintained in an alternating 12-hour light/dark cycle with constant room humidity and temperature. The experiment began at postnatal day 4 (P4), and continued through P32. We assessed and monitored the condition of rat pups on a weekly basis regularly and twice per day in a daily basis especially for the seven days after modeling. All surgery was performed under inhaled anesthesia that was a mixture of halothane and 2:1 nitrous oxide: oxygen, and all efforts were made to minimize suffering. In this model, humane endpoints were used. Humane endpoints were composed of body weight growth (1: slower growth than normal rats, 2: growth arrest, 3: weight loss), responsiveness (1: delayed but appropriate response, 2: delayed and null response, 3: no response), and appearance (1: rough hair coat, 2: porphyrin staining, 3: sustained abnormal posture or dilated pupil). We assessed and monitored the condition of animals on a weekly basis regularly and twice per day in a daily basis especially for the seven days after modeling.

Based on the above scoring system, total scores over than 5 or scores over than 3 in a single category were determined as humane endpoint. To minimize the suffering of the rats, we fed the rats those showed slower weight gain than normal rats, with supplementary artificial milk at least more than four times per day. Euthanasia was planned to be performed via a deep pentobarbital (Entobar, Hanlim Pharmaceutical Co., Ltd., Seoul, Korea) anesthesia (60 mg/kg, intraperitoneal), however, no rats in the present study met the humane endpoint before sacrifice.

In P4 rat pups, IVH was induced by IC injection of a total of 200 μl of fresh maternal whole blood (100 μl each into the right and left ventricles) under stereotactic guidance (Digital Stereotaxic Instrument with Fine Drive, MyNeurolab, St. Louis, MO, USA) as described previously[9]. Normal control rats received a sham operation without IC blood injection (NC, n = 15). After procedure, rat pups were then returned to their dams. At P5 (1 day after IVH), severe IVH simulating grade III or IV IVH in human infants[24] was confirmed by brain magnetic resonance imaging (MRI) and IVH rat pups showed minimal or non-visible IVH on the brain MRI were excluded. At P6, IVH rats were randomly allocated into five experimental groups: IVH control (IC, n = 15), IVH with IV mannitol (IC+man, n = 14), IVH with IV MSCs (IMV, n = 13), IVH with IV MSCs and mannitol (IMV+man, n = 17), and IVH with IC MSCs (IMC, n = 19). The IMC group received 1×10^5^ human UCB-derived MSCs in 10 μl of normal saline (NS) into the right ventricle under stereotactic guidance, as previously reported[9]. An equal volume of NS in the right ventricle was given in the ICgroup. Rat pups in the IMV group received 5×10^5^ human UCB-derived MSCs in 50 μl of NS in the right jugular vein. Immediately before IV administration of MSCs or NS, 10 μl/g body weight of 1.1 mol/l mannitol (maintained at 4°C) was slowly infused through the left jugular vein of the IC+man and IMV+man groups. IC and IMV groups received an equal volume of NS (also maintained at 4°C). The IVH induction and transplantation procedures did not cause any mortality. Follow-up brain MRI was performed 7 (P11) and 28 (P32) days after inducing severe IVH. A sensorimotor test (rotarod test) was performed from 26 (P30) to 28 (P32) days after inducing severe IVH. Animals were weighed daily and euthanized at P32 under deep ketamine/xylazine anesthesia. Whole brain tissue samples were prepared as previously reported [9].

### In Vivo MRI and Assessment of the Ventricle to Whole Brain Volume Ratio

Brain MRI was performed at P5 (1 day after inducing IVH) to confirm severe IVH, and follow-up MRIs were performed at P11 and P32 to monitor the development of PHH. MRIs were performed with a 7.0-Tesla MRI system (Bruker-Biospin, Fällanden, Switzerland)[9, 19]. MRI examination was performed under inhaled anesthesia with 1.5–2% isoflurane in oxygen-enriched air. MRI was performed with a 20-cm gradient set capable of providing a rising time of 400 mTm-1. Images were acquired for 12 coronal slices that were 1.0 mm thick with no interslice gap. For T2-weighted images (T2WI), a fast spin echo sequence was used to acquire images with a repetition time (TR) = 3,000 ms, time to echo (TE) = 60 ms, field of view (FOV) = 25.6 mm x 25.6 mm, matrix size = 256 x 256, and number of excitations (NEX) = 12. One MRI session took 30 minutes per pup on average. The ventricle to whole brain volume ratio, which represents the extent of PHH, was calculated for each rat pup by an examiner blinded to the study group as previously reported [9, 19].

### Functional Behavioral Test

To evaluate the sensorimotor function of rats, the rotarod test was performed three times a day at P30–32 consecutively [9, 19]. The average latency to fall from three trials was used as the final result. The rotation speed of the treadmill was accelerated from 4 to 40 rpm over 100 seconds for a maximum of 3 minutes. Behavioral test was conducted by evaluators who were blind to group assignments.

### TUNEL Assay

Cell death was evaluated with the immunofluorescent terminal deoxynucleotidyltransferase-mediated deoxyuridine triphosphate nick end labeling (TUNEL) technique (kit S7110 ApopTag, Chemicon, Temecula, CA, USA) in the periventricular white matter as described in our previous reports[9, 19]. Briefly, paraffin section slides were deparaffinized, rehydrated, digested at room temperature for 15 minutes, and washed in PBS. Sections were then incubated with equilibration buffer for 1 minute and immediately incubated with working strength TdT enzyme. Fluorescein isothiocyanate (FITC)-labeled anti-digoxigenin conjugate was applied to the sections. The immunofluorescent terminal deoxynucleotidyltransferase-mediated deoxyuridine triphosphate nick end labeling (TUNEL) technique (kit S7110 ApopTag, Chemicon, Temecula, CA, USA) was applied according to the manufacturer’s protocol to assess cell death in the periventricular white matter. Three coronal sections (+0.95 mm to -0.11 mm/Bregma) were selected from each brain and three random, non-overlapping fields in the periventricular area, including the corpus callosum and caudate nucleus, from each section were assessed. TUNEL-positive nuclei in each selected field were counted by an evaluator blinded to the groups.

### Immunohistochemistry

Immunohistochemical evaluation of reactive gliosis (neuronal specific glial fibrillary acidic protein [GFAP]), reactive microglia (ED-1), and myelination (myelin basic protein [MBP]) in the periventricular area was performed on deparaffinized 4-μm thick brain sections as described in our previous reports [9, 19]. Breifly, brain coronal sections were incubated with the following primary antibodies: GFAP (rabbit polyclonal, Dako, Glostrup, Denmark; 1:1000 dilution) and MBP (rabbit polyclonal, Abcam, Cambridge, MA, USA; 1:1000 dilution) or ED-1 (mouse monoclonal, Millipore, Concord Road, MA, USA; 1:100 dilution) as previously reported[9, 19]. Three coronal sections (+0.95 mm to -0.11 mm/Bregma) from each brain were stained. From each section, three random non-overlapping fields in the periventricular area was selected for evaluation. The immunofluorescent intensity of GFAP or MBP staining was measured in the randomly selected fields in the left and right periventricular area using Image J software (National Institutes of Health, USA) by an evaluator blinded to the experimental groups. The number of ED-1 positive cells was counted in the randomly selected fields by an investigator blinded to the group assignments.

### Corpus Callosum Thickness

The thickness of the corpus callosum was measured in the midline at the level of the medial septum area on hematoxylin and eosin-stained coronal paraffin sections (+0.95 mm to -0.11/Bregma), as describe in our previous reports [9].

### Enzyme-linked Immunosorbent assay (ELISA)

Frozen brain tissue samples from the periventricular area were homogenized and centrifuged at 8,000 g for 20 minutes at 4°C. The protein content in the supernatant was measured using the Bradford method with bovine serum albumin (Sigma-Aldrich) as a standard. Interleukin (IL)-1α, IL-1β, IL-6, and tumor necrosis factor (TNF)-α concentrations in homogenates of periventricular brain tissue were measured with the Milliplex MAP ELISA Kit according to the manufacturer’s protocol (Millipore, Billerica, MA, USA)[9].

### Blood-brain barrier permeability

To test changes in the BBB permeability after inducing severe IVH at P4, 2% Evans blue (Sigma-Aldrich, St. Louis, MO, USA) in saline (4 mg/kg of body weight) was injected intraperitoneally to IC (n = 8) and NC (n = 6) rat pups at P5. At P6, the brains were removed, frozen in liquid nitrogen, and stored at −80°C for later analyses. The brain tissue was homogenized and centrifuged (30 min, 15,000 rcf, 4°C). The supernatant was added to an equal volume of trichloroacetic acid. After incubating overnight at 4°C, the solution was centrifuged (30 min, 15,000 rcf, 4°C). The absorbance of the supernatant at 610 nm was measured by a spectrophotometer (xMarkTM, Bio-Rad, Hercules, CA, USA) and the concentration of Evans blue stain was determined according to a standard curve. The results are presented as (μg of Evans blue stain)/(g of brain tissue).

### Confirmation of Donor Cells

Before MSCs were transplanted at P6, donor cells were labeled with microparticles of iron oxide (MPIO), which can be detected as low signal density areas in a T2*W brain MRI. To confirm the presence and localization of donor cells, an in vivo follow-up T2*W brain MRI was performed at P32 (26 days after MSC transplantation).

### Statistical Analyses

Data are expressed as the mean ± standard error of the mean. For continuous variables, statistical comparison between groups was performed by one-way analysis of variance and Tukey’s post hoc analysis. All of the data were analyzed using SPSS version 18.0 (IBM, Chicago, IL, USA). *P* < 0.05 was considered statistically significant.

## Results

### Survival and body weight

Survival rate (100%, 87%, 86%, 100%, 88% and 100% in the NC, IC, IC+man, IMV, IMV+man, and IMC groups, respectively) and body weight did not differ significantly among study groups (S1 Fig).

### Serial Brain MRI

**Fig 1:**
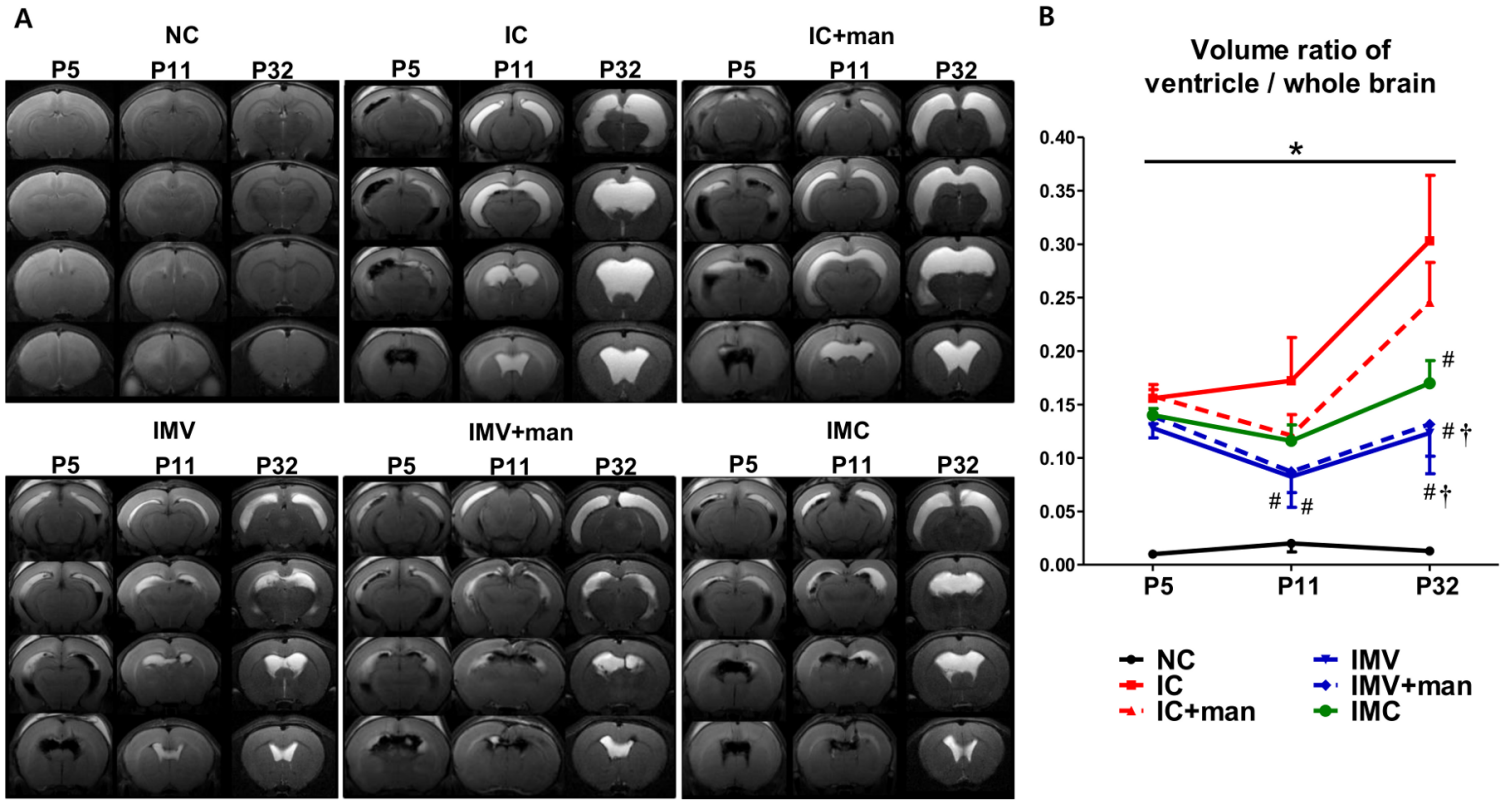
Vetricular dilatation and its progression after severe intraventricular hemorrhage (IVH). A, Representative serial brain MRIs from each group 1, 7, and 28 days after inducing IVH (P5/P11/P32). B, The ventricle to whole brain volume ratio as measured by MRI. Data are expressed as mean ± SEM. NC, normal control rats; IC, IVH control rats; IC+man, IVH control rats+mannitol; IMV, IVH with intravenous transplantation of human umbilical cord blood (UCB)-derived mesenchymal stem cells (MSCs); IMV+man, IVH with intravenous transplantation of human UCB-MSCs+mannitol; IMC, IVH with intracerebroventricular transplantation of human UCB-MSCs. * *P* <0.05 vs. NC, # *P* <0.05 vs. IC, †*P* <0.05 vs. IC+man

Fig 1A displays serial brain MRIs from each study group performed at 1, 7, and 28 days after inducing IVH (P5, P11, and P32, respectively). The ventricular dilatation severity, presented as a ratio of the ventricle to whole brain volume, 1 day after inducing IVH (P5) was significantly higher in the IVH induced groups than in the NC group but did not differ significantly among the experimental groups (15.5±0.8%, 15.8±1.0%, 13.8±0.9%, 13.9±0.7%, 13.1±0.6%, and 14.1±0.3% in the IC, IC+man, IMV, IMV+man, and IMC groups, respectively) (Fig 1B). On follow-up MRIs done at P11 and P32 after infusing vehicle or MSCs with and without mannitol at P6, the IC group had active progression of ventriculomegaly (30.3±6.1% at P32), which was significantly attenuated in the IMV, IMV+ man, and IMC groups (12.3±3.8%, 13.2±3.0%, and 18.7±2.6%, respectively, at P32) but not in the IC+man group (25.6±3.7%). These results suggest that both IV and IC MSC transplantation were equally effective in attenuating PHH after severe IVH, but mannitol neither improved PHH nor enhanced protection by IV MSCs.

### Functional Behavior Tests

**Fig 2:**
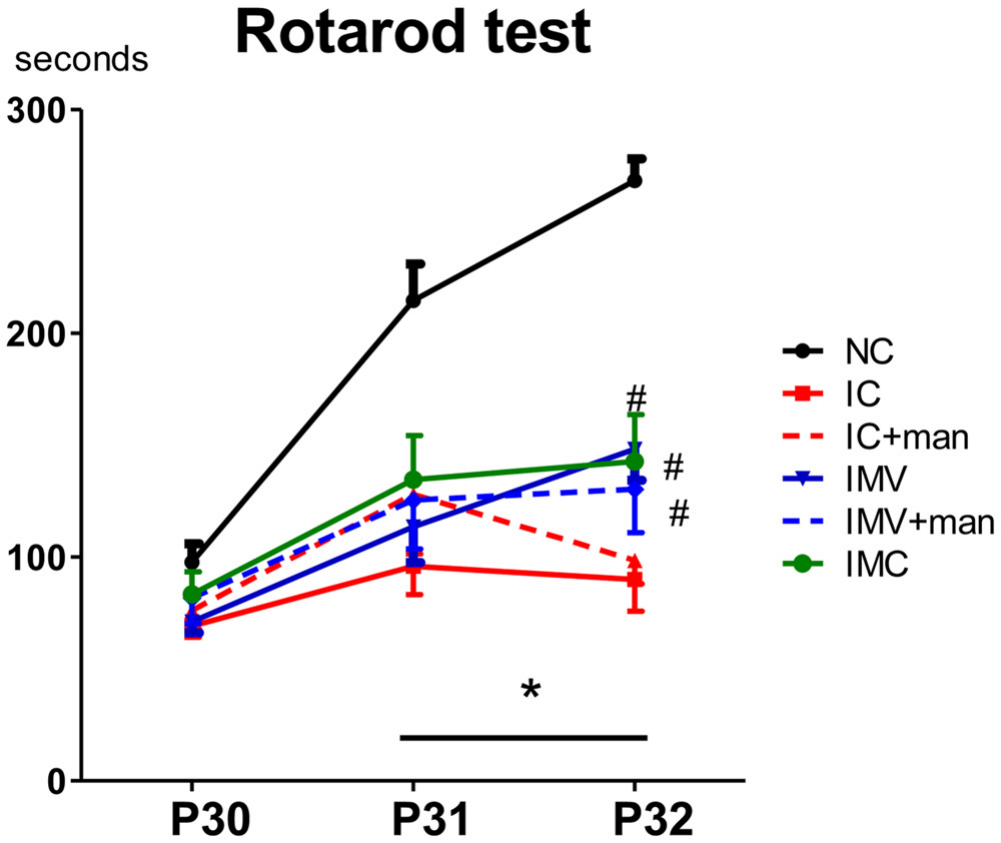
Sensorimotor function after severe IVH in newborn rats. Sensorimotor functional outcomes on the rotarod test. Data are expressed as mean ± SEM. NC, normal control rats; IC, IVH control rats; IC+man, IVH control rats+mannitol; IMV, IVH with intravenous transplantation of human UCB-MSCs; IMV+man, IVH with intravenous transplantation of human UCB-MSCs+mannitol; IMC, IVH with intracerebroventricular transplantation of human UCB-MSCs. * *P* <0.05 vs. NC, # *P* <0.05 vs. IC

To assess the sensorimotor function, the rotarod test was performed at P30, P31, and P32. Although results at P30 did not differ significantly between study groups, the IC group had a significantly shorter latency to fall than did the NC group at P31 and P32 (Fig 2). This impaired function in the IC group was significantly improved in both the IMV and IMC groups. Mannitol neither attenuated rotarod test impairments nor enhanced the improvements due to transplanting IV MSCs after severe IVH.

### Cell Death and Reactive Gliosis

**Fig 3:**
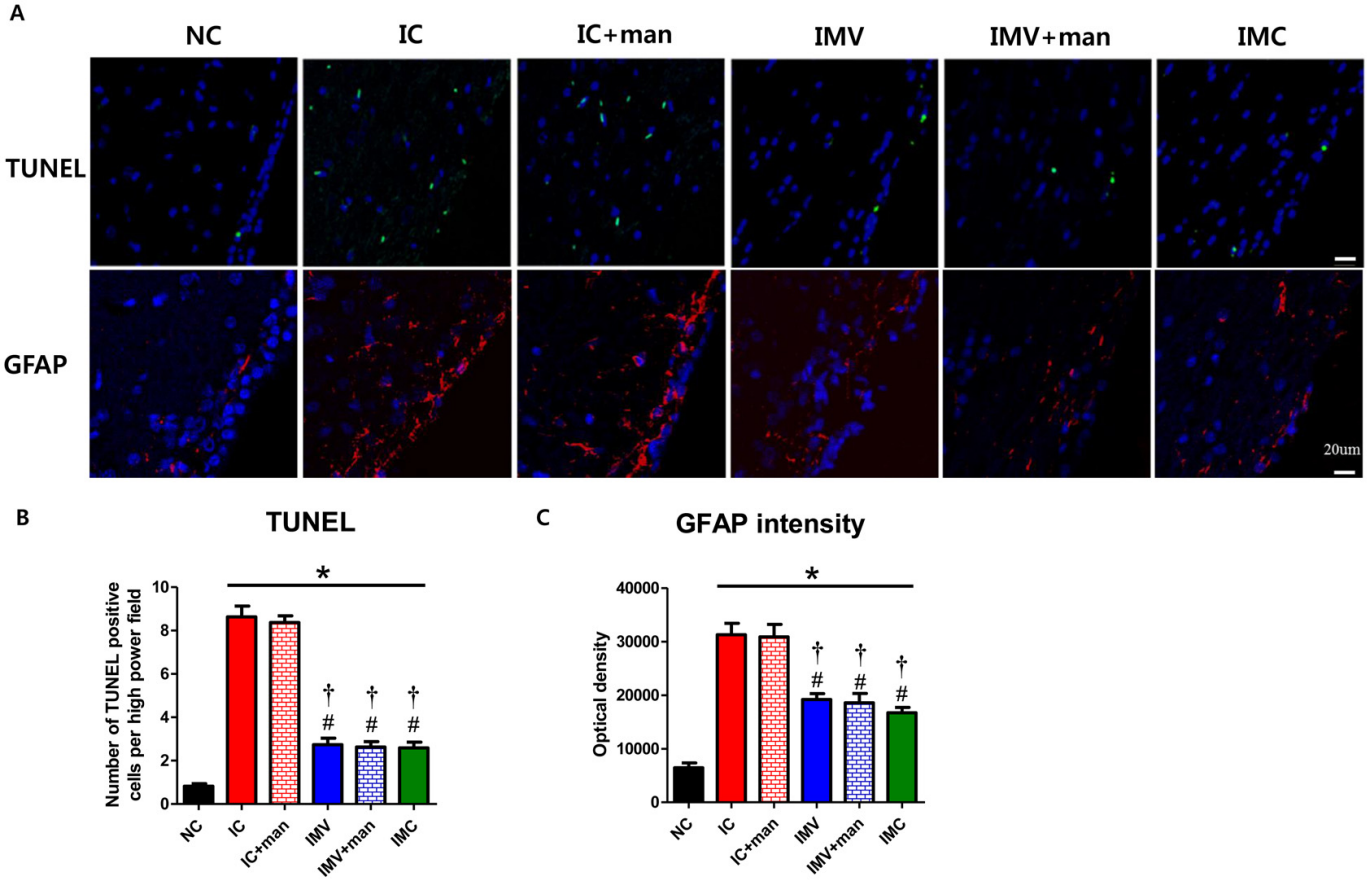
Cell death and reactive gliosis induced by severe IVH in newborn rats. A, Representative immunofluorescence photomicrographs of the periventricular area with staining for TUNEL (green) and glial fibrillary acidic protein (GFAP) (red), and DAPI (blue) (original magnification; x400, scale bars; 20 μm). Average number of TUNEL-positive cells (B) and average density of GFAP staining (C) in the periventricular area. Data are expressed as mean ± SEM. NC, normal control rats; IC, IVH control rats; IC+man, IVH control rats+mannitol; IMV, IVH with intravenous transplantation of human UCB-MSCs; IMV+man, IVH with intravenous transplantation of human UCB-MSCs+mannitol; IMC, IVH with intracerebroventricular transplantation of human UCB-MSCs. * *P* <0.05 vs. NC, # *P* <0.05 vs. IC, †*P* <0.05 vs. IC+man.

To determine the extent of periventricular brain injury after severe IVH, the number of TUNEL-positive cells and density of GFAP-positive cells were assessed by immunohistochemistry of periventricular brain tissue at P32. The number of TUNEL-positive cells and density of GFAP-positive cells in the IC group were significantly higher than in the NC group (Fig 3). The abnormalities observed in the IC group were significantly improved in the IMC, IMV, and IMV+man groups but not in the IC+man group. The extent of attenuation did not differ significantly among the IMC, IMV, and IMV+man groups, indicating that mannitol co-infusion did not augment the benefits of administering IV MSCs after severe IVH.

### Myelination and Corpus Callosum Thickness

**Fig 4:**
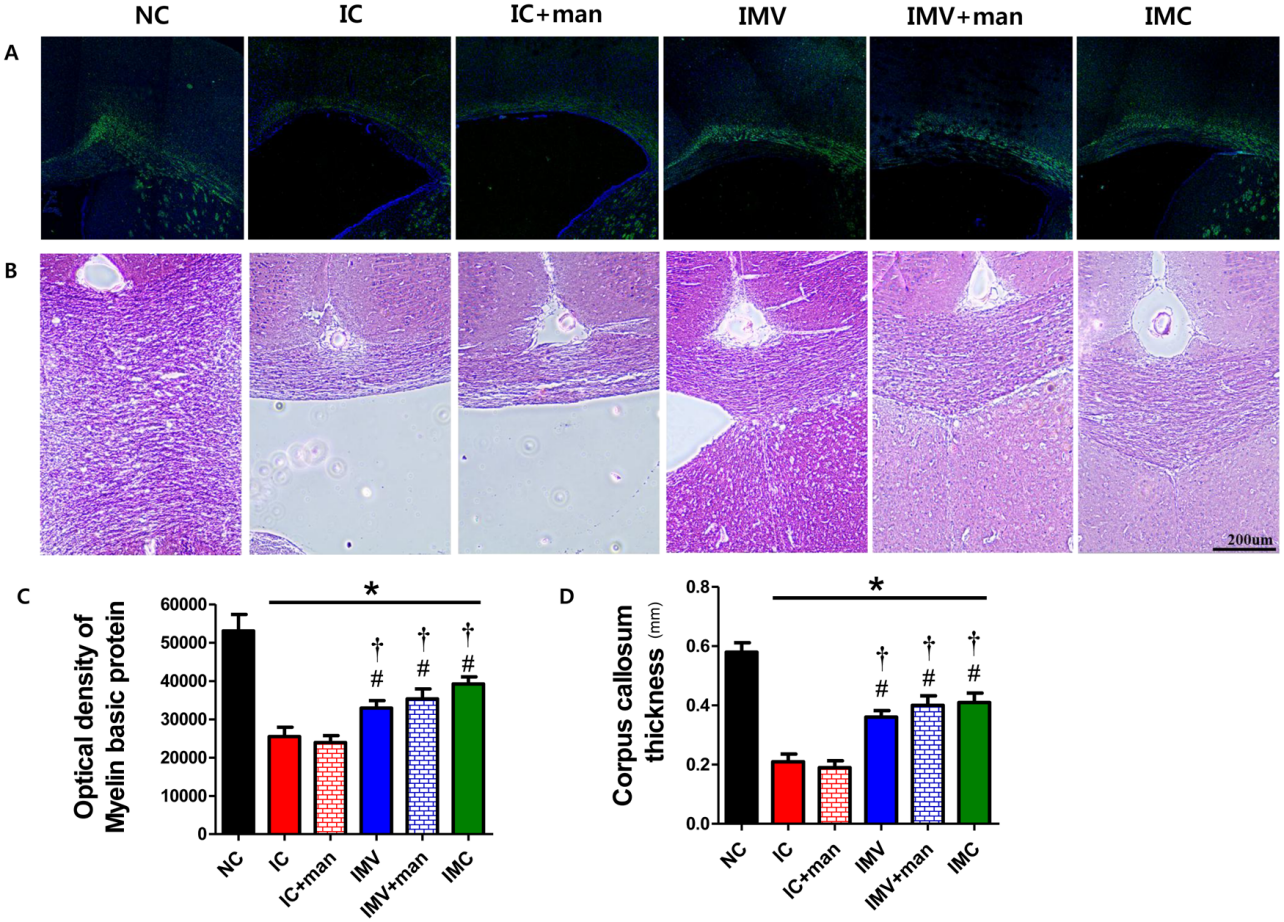
Delayed myelination and corpus callosum thinning induced by posthemorrhagic hydrocephalus in newborn rats. A, Representative immunofluorescence photomicrographs of the periventricular area in each group with staining for myelin basic protein (MBP) (green) and DAPI (blue). B, Average MBP density in each group. C, Representative optical photomicrographs of the corpus callosum stained with hematoxylin and eosin (original magnification; x100, scale bars; 200 μm). D, Corpus callosum thickness in each group. Data are expressed as mean ± SEM. NC, normal control rats; IC, IVH control rats; IC+man, IVH control rats+mannitol; IMV, IVH with intravenous transplantation of human UCB-MSCs; IMV+man, IVH with intravenous transplantation of human UCB-MSCs+mannitol; IMC, IVH with intracerebroventricular transplantation of human UCB-MSCs. * P <0.05 vs. NC, # P <0.05 vs. IC, †P <0.05 vs. IC+man.

Myelination in the periventricular area was estimated by MBP immunostaining at P32. The optical density of MBP, indicative of the extent of myelination, was significantly lower in the IC group than in the NC group. This impaired myelination was significantly improved in the IMC, IMV, and IMV+man groups but not in the IC+man group (Fig 4A and 4C).

As ventricular dilatation progressed, the anterior corpus callosum (measured at the midline of coronal sections at the level of the medial septum) at P32 was significantly thinner in the IC group than in the NC group (Fig 4B and 4D). This compression of the periventricular corpus callosum induced by PHH after severe IVH was significantly improved in the IMC, IMV, and IMV+man groups but not in the IC+man group.

### Inflammation in the brain

**Fig 5:**
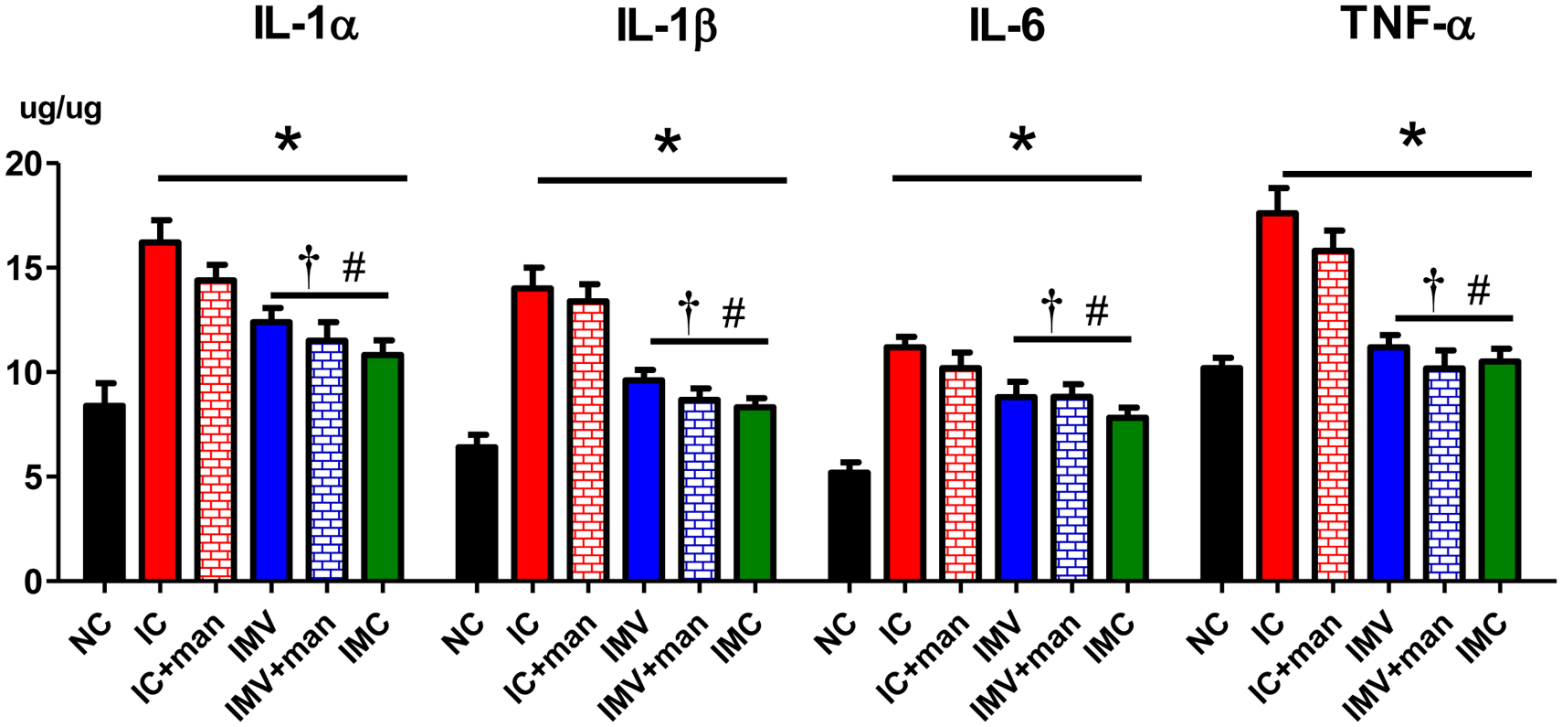
Inflammatory cytokines in periventricular brain tissue at P32. The levels of inflammatory cytokines includingIL-1α, IL-β, IL-6, and TNF- α in brain homogenates from the periventricular area at P32 were measured. Data are expressed as mean ± SEM. NC, normal control rats; IC, IVH control rats; IC+man, IVH control rats+mannitol; IMV, IVH with intravenous transplantation of human UCB-MSCs; IMV+man, IVH with intravenous transplantation of human UCB-MSCs+mannitol; IMC, IVH with intracerebroventricular transplantation of human UCB-MSCs. * P <0.05 vs. NC, # P <0.05 vs. IC, †P <0.05 vs. IC+man.

**Fig 6:**
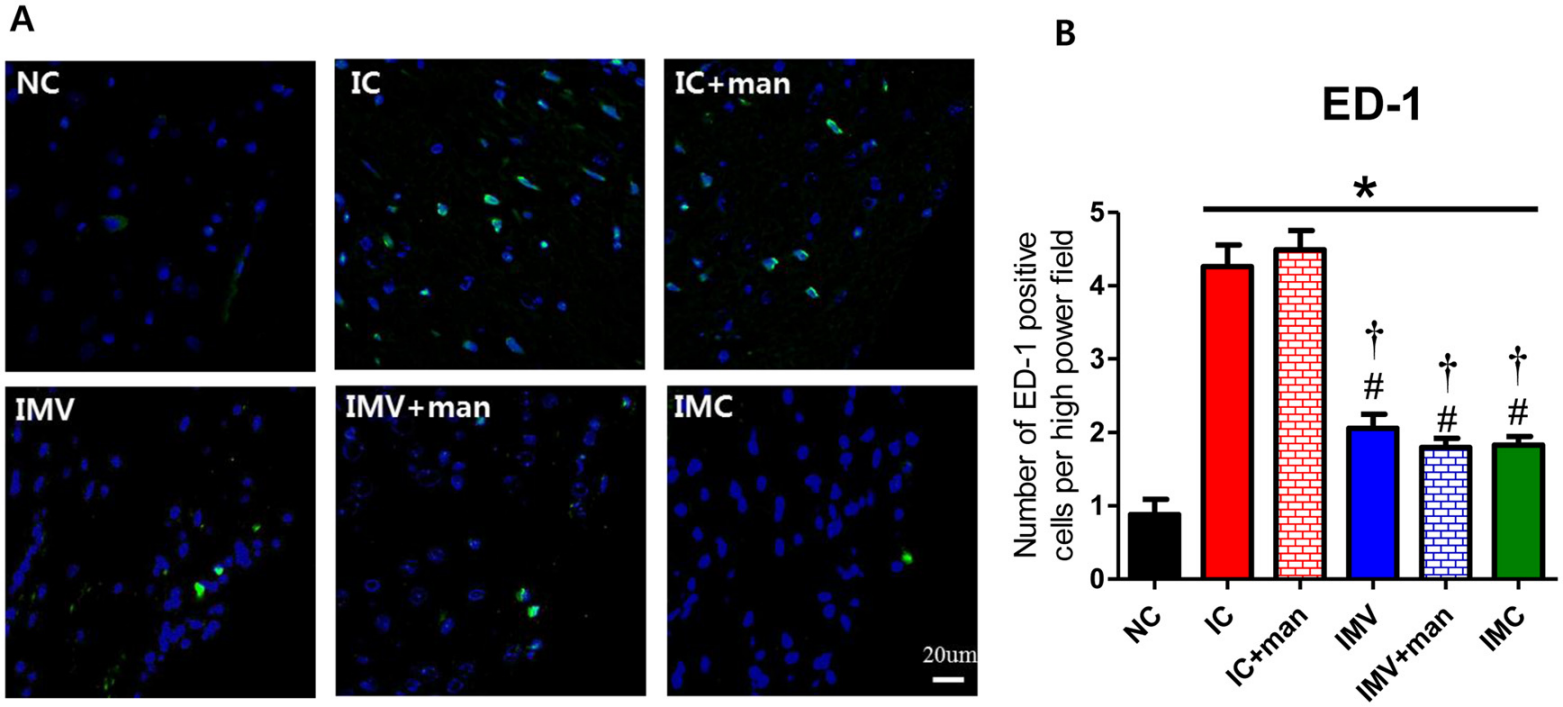
Active macrophages in the periventricular brain tissue after severe IVH in newborn rats. A, Representative immunofluorescence photomicrographs of the periventricular area with staining for ED-1 (green) and DAPI (blue) (original magnification; x400, scale bars; 20 μm). B, Average number of ED-1 positive cells in the periventricular area. Data are expressed as mean ± SEM. NC, normal control rats; IC, IVH control rats; IC+man, IVH control rats+mannitol; IMV, IVH with intravenous transplantation of human UCB-MSCs; IMV+man, IVH with intravenous transplantation of human UCB-MSCs+mannitol; IMC, IVH with intracerebroventricular transplantation of human UCB-MSC. * P <0.05 vs. NC, # P <0.05 vs. IC, †P <0.05 vs. IC+man.

To determine whether transplanted MSCs attenuated brain inflammation induced by severe IVH, we analyzed levels of inflammatory cytokines including IL-1α, IL-1β, IL-6, and TNF-α in periventricular brain tissue homogenates and the number of ED-1 positive cells in brain coronal sections at P32. Inflammatory cytokine and ED-1 positive cell levels in the periventricular brain tissue were significantly higher in the IC group than in the NC group. These increased inflammatory responses were significantly attenuated in the IMC, IMV, and IMV+man groups but not in the IC+man group (Figs 5 and 6).

### BBB Permeability

The Evans blue concentration in brain tissue at P6 was significantly higher in the IC group than in the NC group, indicating increased BBB permeability after inducing severe IVH at P4 (S2 Fig).

### Confirmation of Donor Cells

T2-weighted imaging at P11 revealed areas of low signal intensity, reflecting the presence of transplanted, MPIO-tagged MSCs in the bilateral periventricular area of the IMC, IMV, and IMV+man groups but not the NC, IC, or IC+man groups[9, 19] (S3 Fig).The area of low signal intensity seemed larger in the IMC group than in the IMV and IMV+man groups. These findings suggest that the MSC delivery efficiency is better with IC than with IV infusion and that mannitol co-infusion does not augment the permeability of IV-infused MSCs through the already leaky BBB after severe IVH.

## Discussion

Developing an appropriate animal model to simulate clinically severe IVH and the ensuing PHH in premature infants is essential to understand the nature of the pathogenesis of this condition and for testing the efficacy of new therapies. In this study, we used P4 rats, which are more immature than P7 rats, because severe IVH is more common with increasing immaturity[2]. We consistently induced PHH after severe IVH by injecting 100 ul of fresh maternal blood into each lateral ventricle with a tolerable 13% mortality rate[9]. Our data showing impaired behavioral function and histologic abnormalities besides PHH after severe IVH indicate that our P4 newborn rat pup model is suitable and appropriate for researching severe IVH and the ensuing PHH in very preterm infants.

The therapeutic efficacy of IV transplantation of MSCs is limited by the ability of these large cells to pass through an intact BBB, which is a key factor to mediate recovery. However, rat pups at P6 might have an ontogenically immature BBB[25, 26], which might be enhanced by brain inflammation[27, 28]. In the present study, the BBB permeability 2 days after inducing severe IVH at P6 was significantly higher than in the NC group. Although 5-times more MSCs were transplanted by the IV route, more MPIO-tagged MSCs engrafted to the periventricular injury site with IC transplantation, as detected by brain MRI at P12[29]. Nonetheless, both local IC and systemic IV MSC administration were equally effective in protecting against PHH, impaired sensorimotor function, and brain injury including inflammation, cell death, reactive gliosis, and delayed myelination after severe IVH. Taken together, our data suggest that despite the inferior delivery efficiency of IV transplantation, both IV and IC transplantation of MSCs had equally protected against severe IVH. Clinically >90% of IVH occurs within the most vulnerable period of 3 days after birth[10], so some critically ill preterm infants might not tolerate the more invasive local IC infusion of MSCs. The less invasive IV route could thus be a safe and effective alternative method to administer MSCs in some clinically unstable preterm infants with severe IVH.

After an IVH insult, a cascade of inflammatory responses affects not only the development of PHH but also the extent of brain injury[30]. In previous studies, we showed that the neuroprotective effects of transplanting MSCs after severe IVH might be primarily mediated by their paracrine anti-inflammatory effects rather than by their regenerative capabilities[9]. Neurotrophic factors and cytokines, such as vascular endothelial growth factor, brain derived neurotrophic factor, glial cell line derived neurotrophic factor, and IL-10, secreted by engrafted MSCs might mediate anti-inflammatory and anti-apoptotic effects and stimulate endogenous cell proliferation and differentiation after severe IVH[31–35]. We are currently working to identify the trophic factors secreted by transplanted MSCs that might mediate these beneficial effects[36–39].

Improving functional sensorimotor outcome is important for future clinical translation of MSC transplantation for severe IVH in premature infants. The extent of PHH, abnormal histology, and impaired functional sensorimotor outcome were clearly related in this study. The significantly improved rotarod tests with both IV and IC MSC transplantation might be attributed to preventing PHH and attenuating the inflammatory responses after severe IVH. Moreover, improved rotarod test results at P31 and P32 with both IV and IC MSCs transplantation at P6 imply that the transplant-mediated neuroprotective effects might persist into human adolescence.

In the present study, injecting a BBB permeabilizer (mannitol) alone did not have any neuroprotective effects after severe IVH[40]. Moreover, mannitol co-infusion did not facilitate the migration of IV-administered MSCs across the BBB, nor did it augment the neuroprotective effects of MSCs against severe IVH. Our data suggest that manipulating the already leaky BBB with mannitol co-infusion does not facilitate the entry of IV-administered MSCs into the injured brain and, thus, fails to enhance neuroprotection after severe IVH.

The optimal dose of MSCs delivered either by a local IC or systemic IV approach also needs to be addressed for clinical translation. In the present study, the MSC dose given systemically was arbitrarily selected to be 5 times the local dose based on previous data showing that transplanting 5-fold more MSCs by systemic intraperitoneal administration than by local intratracheal administration resulted in a lower engraftment efficacy in hyperoxic lung injury[20]. Since we showed equal therapeutic efficacy with both local IC and systemic IV MSC transplantation in this study, further studies will be necessary to determine the optimal MSC dose for each route to maximize neuroprotection after severe IVH.

In summary, although local IC delivery results in better delivery efficacy than does systemic IV delivery, both IV and IC transplantation of MSCs had equal therapeutic efficacy by significantly attenuating PHH, abnormal behavioral tests, and brain injury after severe IVH regardless of simultaneous mannitol co-infusion. Our data suggest that the more practical and less invasive method of systemic IV MSC delivery might be a good alternative to local IC transplantation in some clinically unstable preterm infants with severe IVH.

## Supporting Information

S1 Fig Growth in weight in each group. NC, normal control rats; IC, IVH control rats; IC+man, IVH control rats+mannitol; IMV, IVH with intravenous transplantation of human UCB-MSCs; IMV+man, IVH with intravenous transplantation of human UCB-MSCs+mannitol; IMC, IVH with intracerebroventricular transplantation of human UCB-MSC. Data are expressed as mean ± SEM. * P <0.05 vs. Normal.
(TIF)

S2 Fig Increased blood-brain barrier permeability after intraventricular hemorrhage (IVH). A, Appearance of rat pups and harvested brains at postnatal day (P) 6 after peritoneal injection of Evans blue dye at P5. B, Concentration of Evans blue in brain tissue homogenates from normal and IVH-induced rats at P6. Data are expressed as mean ± SEM. * P <0.05 vs. Normal.
(TIF)

S3 Fig Localization of donor cells. Localization of grafted human umbilical cord blood (UCB)-derived mesenchymal stem cells (MSCs) that were tagged with micron-sized paramagnetic iron-oxide (MPIO) particles. The presence of donor cells injected intravenously or intracerebroventricularly at P5 was confirmed as low signal-intensity by T2* MRI at P11 in the periventricular areas reflecting MPIO.
(TIF)
