# Supplementary figures and images for: Optimal Route for Mesenchymal Stem Cells Transplantation after Severe Intraventricular Hemorrhage in Newborn Rats
Source: PLoS One. 2015 Jul 24;10(7):e0132919. doi: 10.1371/journal.pone.0132919 (PMC4514759; doi:10.1371/journal.pone.0132919)

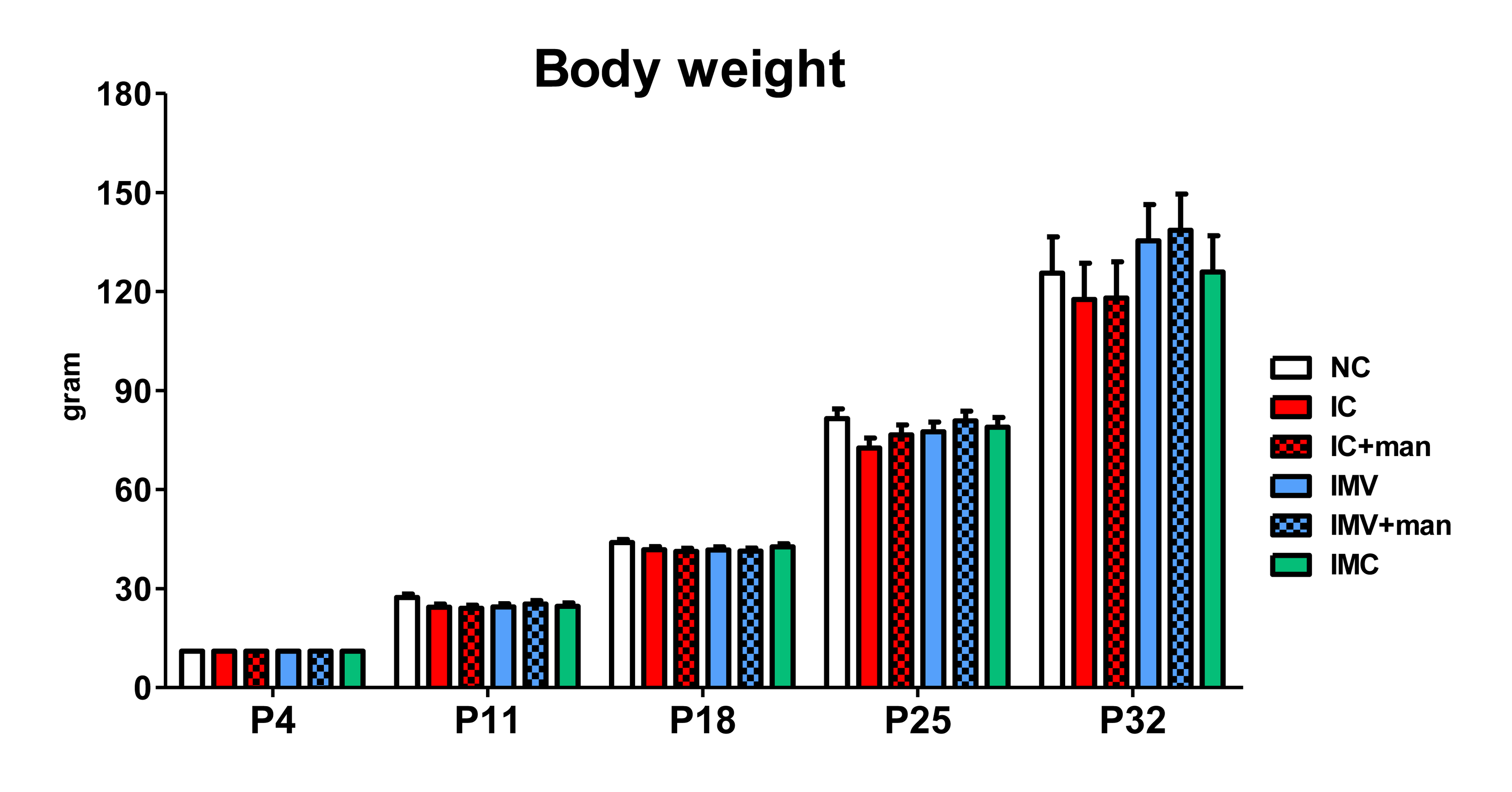

Supplement: S1 Fig — NC, normal control rats; IC, IVH control rats; IC+man, IVH control rats+mannitol; IMV, IVH with intravenous transplantation of human UCB-MSCs; IMV+man, IVH with intravenous transplantation of human UCB-MSCs+mannitol; IMC, IVH with intracerebroventricular transplantation of human UCB-MSC. Data are expressed as mean ± SEM. * P <0.05 vs. Normal. (TIF) [file pone.0132919.s001.tif]

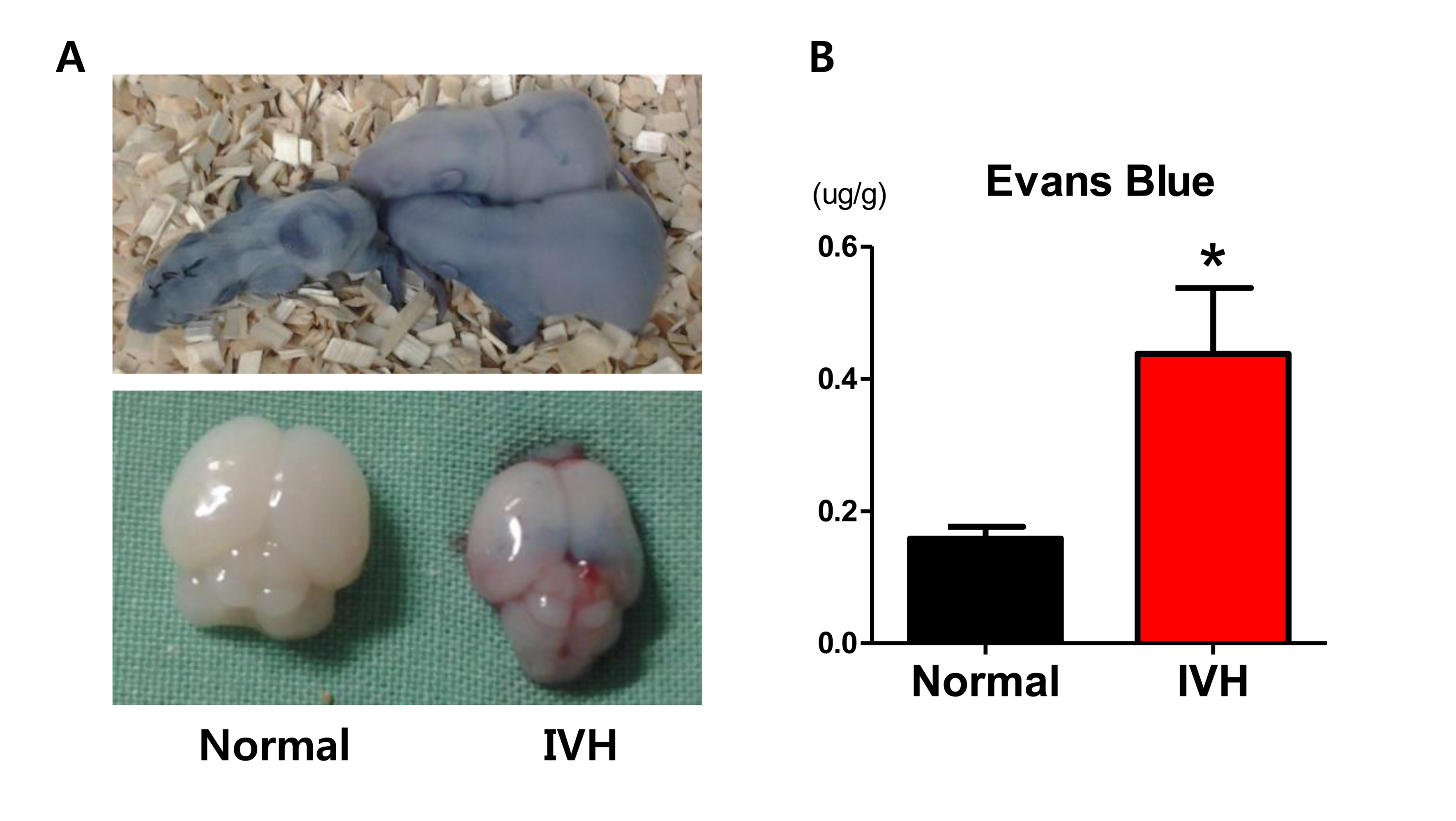

Supplement: S2 Fig — A, Appearance of rat pups and harvested brains at postnatal day (P) 6 after peritoneal injection of Evans blue dye at P5. B, Concentration of Evans blue in brain tissue homogenates from normal and IVH-induced rats at P6. Data are expressed as mean ± SEM. * P <0.05 vs. Normal. (TIF) [file pone.0132919.s002.tif]

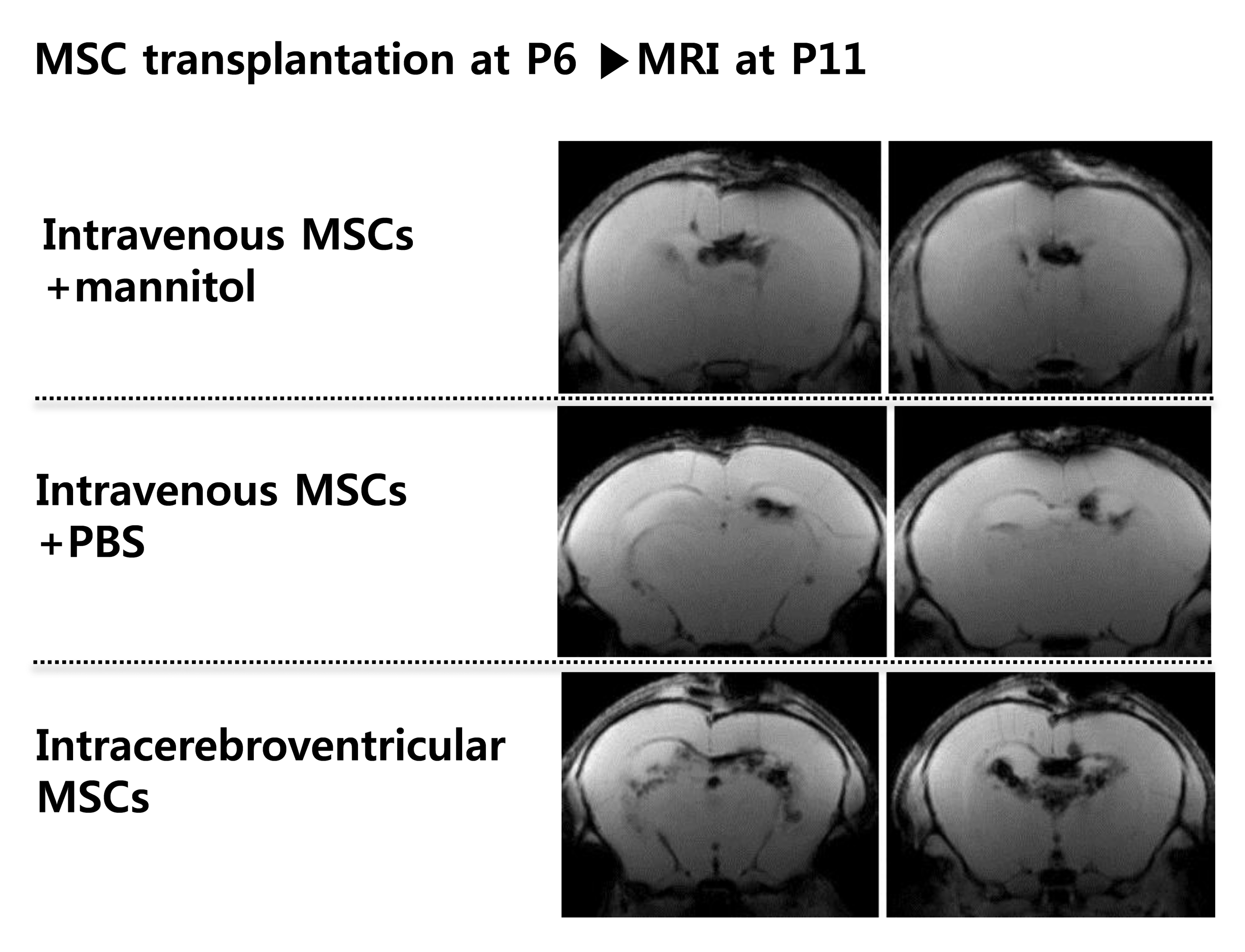

Supplement: S3 Fig — Localization of grafted human umbilical cord blood (UCB)-derived mesenchymal stem cells (MSCs) that were tagged with micron-sized paramagnetic iron-oxide (MPIO) particles. The presence of donor cells injected intravenously or intracerebroventricularly at P5 was confirmed as low signal-intensity by T2* MRI at P11 in the periventricular areas reflecting MPIO. (TIF) [file pone.0132919.s003.tif]
